# Differences between expert reported and patient reported burden of disease rankings

**DOI:** 10.1038/s41598-021-04070-5

**Published:** 2022-01-18

**Authors:** Damien S. E. Broekharst, Sjaak Bloem, Edward A. G. Groenland, W. Fred van Raaij, Michel van Agthoven

**Affiliations:** 1 Center for Marketing and Supply Chain Management, Nyenrode Business University, Breukelen, The Netherlands; 2 Janssen-Cilag B.V., Johnson and Johnson, Breda, The Netherlands; 3 Department of Social Psychology, Tilburg University, Tilburg, The Netherlands

**Keywords:** Health care economics, Health policy, Health services, Public health

## Abstract

Many attempted to develop burden of disease rankings for the purpose of resource allocation, priority setting, cost-effectiveness evaluation, and service development in healthcare. As this proved difficult the World Health Organization commissioned expert panels to develop internally consistent burden of disease rankings. Although these rankings provide valuable insight in the biomedical burden of different diseases, they do not yet provide insight in the psychological burden of different diseases experienced and reported by patients on a daily basis. Since expert reported and patient reported burden of disease could differ, deviations between expert reported and patient reported burden of disease rankings are likely. To explore how these rankings differ, it is important to develop patient reported burden of disease rankings and compare these to expert reported burden of disease rankings. In this study patient reported burden of disease rankings were developed by ranking the subjective health experience of patients. To measure subjective health experience an online questionnaire was administered to a large panel of Dutch citizens. The final sample consisted of 58,490 panel members. This final sample contained 36 diseases and was largely representative of the Dutch population. The data were analysed by using reliability tests, descriptive statistics and Spearman rank-order correlation coefficients. This study shows that expert reported and patient reported burden of disease rankings could differ. Burden of cardiovascular diseases ranks low on patient reported burden of disease rankings, while it ranks higher on expert reported burden of disease rankings. Burden of psychiatric diseases and gastrointestinal diseases ranks high on patient reported burden of disease rankings, while it ranks lower on expert reported burden of disease rankings. Burden of pain diseases ranks high on patient reported burden of disease rankings, while it is still overlooked in expert reported burden of disease rankings. This study suggests that it can be beneficial to develop and utilize patient reported burden of disease rankings in addition to the already existing expert reported burden of disease rankings in order to establish a more comprehensive perspective on burden of disease. This could improve decision-making on resource allocation, priority setting, cost-effectiveness evaluation, and service development in healthcare.

## Introduction

Over the last decades, scientists and policymakers have attempted to develop burden of disease rankings for the purpose of allocating resources, determining priorities, evaluating cost-effectiveness, and developing new services in healthcare^1–6^. Most of these attempts were frustrated by fragmented, incomparable, advocacy-driven and incomplete health information^6^. This led the World Health Organization to entrust several expert panels (e.g., Child Health Epidemiology Reference Group, Malaria Monitoring and Evaluation Reference Group, Foodborne Disease Burden Epidemiology Reference Group) with the development of internally consistent burden of disease rankings^6^. These rankings are developed by estimating and ranking the mortality and disability resulting from different diseases^7^. Although these rankings provide valuable insight in the biomedical burden of different diseases, they do not yet provide insight in the psychological burden of different diseases experienced and reported by patients on a daily basis. Since patient reported burden of disease often differs from expert reported burden of disease, deviations between patient reported and expert reported burden of disease rankings are likely to occur^8–10^. In order to understand how these rankings differ, it is important to develop patient reported burden of disease rankings and discuss how these compare with the usual expert reported burden of disease rankings. One reliable and valid way to develop patient reported burden of disease rankings is by determining and ranking the subjective health experience of patients^11^.

**Figure 1:**
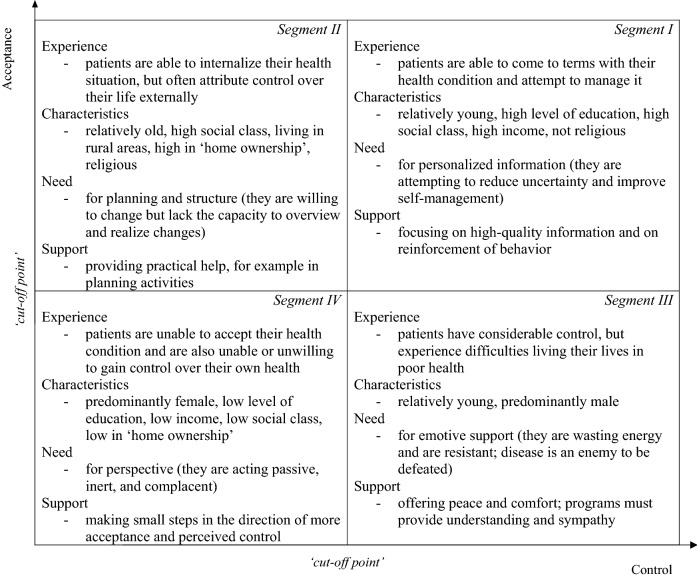
Updated Bloem & Stalpers model of subjective health experience.

Subjective health experience is defined as “an individual’s experience of physical and mental functioning while living his life the way he wants to, within the actual constraints and limitations of individual existence”^12^ [p. 8]. Research shows that the two central determinants of subjective health experience are ‘acceptance’ and ‘perceived control’^11^. ‘Acceptance’ is basically an affective concept: it refers to the extent to which patients are able to experience their health condition as an integral part of their existence^13^. ‘Perceived control’ is basically cognitive in nature: it expresses the extent to which patients believe to be able to exert influence on their condition^14^. Based on these determinants, a model consisting of four segments (see Fig. 1) was developed with each segment describing a particular subjective health experience profile^11^. Patients in segment I are able to come to terms with their health condition and attempt to manage it. Patients in segment II are able to internalize their health situation, but often attribute control over their life externally. Patients in segment III have considerable control, but experience difficulties living their lives in poor health. Patients in segment IV are unable to accept their health condition and are also unable or unwilling to gain control over their own health^11^. As diseases are more incapacitating, a larger proportion of patients accumulates in segment IV of the Bloem & Stalpers model, indicating a higher burden of disease^11^.

Therefore, in this study the proportions of patients in segment IV of the Bloem & Stalpers model are used to determine the burden of different diseases and develop two different patient reported burden of disease rankings^11,15^. The first patient reported burden of disease ranking is developed by including only patients without comorbidities as the usual expert reported burden of disease ranking is also developed for isolated diseases^1–6^. The second patient reported burden of disease ranking is developed by including patients with and without comorbidities as diseases often do not occur in isolation and comorbidities are common^16–18^. After both rankings are developed they are compared with the usual expert reported burden of disease rankings. In doing so, this study addresses three main research questions: (a) How is the burden of different diseases ranked by patients without comorbidities, (b) How is the burden of different diseases ranked by patients with and without comorbidities, and (c) How do these patient reported burden of disease rankings compare to the usual expert reported burden of disease rankings. By answering these questions, this study shows how expert reported and patient reported burden of disease rankings provide different, but uniquely valuable, insight in burden of disease as well as how their complementary use could establish a more comprehensive perspective on burden of disease. This comprehensive perspective could be deployed to improve decision-making on resource allocation, priority setting, cost-effectiveness evaluation and service development in healthcare^1–6^.

## Method

### Procedure, participants and panel characteristics

In order to answer the three main research questions, a questionnaire design was used. The questionnaires were administered online to a sizeable panel of Dutch citizens established by the GfK research agency. The approximately 70,000 panel members were invited by email and were provided with necessary information and a formal request for consent, as is standard policy within the GfK research agency^19^. Only the panel members that consented to the use of their responses in further research, were selected for participation. The final sample used for this study consisted of 58,490 panel members. Before participation, the selected panel members were screened regarding their motivation for participating in research projects, their socio-economic status and their socio-demographic characteristics. The panel members were also screened for duplicate panel memberships. The data used for this research project was gathered in 2016 as part of a large epidemiological population study on health and wellbeing in the Netherlands.

### Questionnaire, items and measurement scales

In order to measure the determinants of subjective health experience, ‘acceptance’ and ‘perceived control’, the questionnaire of Bloem & Stalpers was adopted^11,15^. The Bloem & Stalpers questionnaire was deployed as most health-related quality of life questionnaires do not capture the specific determinants of subjective health experience and are often limited in their scope due to their disease-specific focus or narrow range of broadly defined dimensions. In contrast to the Bloem & Stalpers questionnaire these health-related quality of life questionnaires are also not anchored in a theoretical framework and lack a clear conceptualization and operationalization^11,15^. The six items that comprise the Bloem & Stalpers questionnaire are displayed in Table 1.

**Table 1:** Items of the Bloem & Stalpers questionnaire.

Determinant	Item	M	SD
Acceptance	I am at peace with my health condition	4.85	1.61
	The way in which I am functioning physically and mentally, is acceptable to me	5.15	1.51
	I accept my health condition the way it is	5.06	1.54
Perceived control	I have the feeling that I have grip on my health condition	4.86	1.45
	My health condition is to a great extent in my own power	4.90	1.51
	I have a lot of influence on my health condition	5.29	1.38

The items were measured on a quasimetric (interval) scale ranging from 1 = fully disagree to 7 = fully agree. The questionnaire also included additional items on population characteristics and medically diagnosed diseases. These additional items were measured on nominal scales with dichotomous response categories or ordinal scales using ascending response categories. The additional items included, are described in Table 2.

**Table 2:** Population characteristics and medically diagnosed diseases.

**Population characteristics**	
Socio-economic	Gross annual income, level of education
Socio-demographic	Age, gender, household size, internet use, municipality size, medication use
**Medically diagnosed diseases**	
Oncological diseases	Breast cancer, colon cancer, skin cancer, lung cancer, prostate cancer, other cancer
Neurological diseases	Multiple sclerosis, Parkinson’s disease, migraine, chronic headache
Psychiatric diseases	Depression, ADHD, dementia, Alzheimer’s disease
Gastrointestinal diseases	Chronic constipation, inflammatory bowel disease, obesity
Pulmonary diseases	Asthma, allergic rhinitis, COPD
Rheumatological diseases	Arthrosis, osteoporosis, rheumatism
Cardiovascular diseases	Heart failure, high cholesterol, hypertension
Ophthalmological diseases	Macular degeneration, cataract
Urological diseases	Erectile dysfunction, incontinence
Pain diseases	Lower backpain, chronic pain
Immunological diseases	HIV/Aids, hepatitis C
Endocrinological diseases	Diabetes II
Dermatological diseases	Psoriasis

### Statistical analyses

Factor analysis was conducted in order to condense the three questions on ‘acceptance’ and the three questions on ‘perceived control’ into two independent scales that generated two single scores for every respondent. In addition, the levels of reliability concerning both scales were calculated. Both the ‘acceptance’ scale (*α* = 0.91) as well as the ‘perceived control’ scale (*α* = 0.90) can be considered reliable as the Cronbach’s alpha values of both scales exceeded the minimum acceptable score of 0.70^20,21^. In order to construct the segments, cut-off scores were defined as the mean values of the ‘acceptance’ (M = 5.02, SD = 1.43) and the ‘perceived control’ (M = 5.02, SD = 1.32) scores. The mean values were used as the sample mean is the most accurate point estimate of population mean^22^. This process generated the four main segments of the Bloem & Stalpers model. Based on the proportions of patients in segment IV of the Bloem & Stalpers model a patient reported burden of disease ranking without comorbidities as well as a patient reported burden of disease ranking with and without comorbidities was developed. These rankings were compared to the usual expert reported burden of disease rankings by computing Spearman rank-order correlation coefficients. This coefficient ranges between + 1, which indicates perfect similarity between rankings, and -1, which indicates perfect dissimilarity between rankings^23^. The publicly available expert reported burden of disease rankings used for comparison pertained to the Dutch population and were derived from the Dutch National Institute for Public Health and the Environment^24^. Analyses were performed using IBM SPSS Statistics version 25.

### Ethics declarations

The need for ethics approval is not applicable according to the Dutch Medical Research Involving Human Subjects Act (NWO). All methods were carried out in accordance with relevant guidelines and regulations. The GfK database was also acquired in line with the Dutch laws, rules and regulations on research ethics as is explicitly explained in their research policy (https://www.gfk.com/).

### Consent to participate

Written informed consent was obtained from all participants.

## Results

### Sample description

Of the 58,490 panel members in the final sample 39,131 had no medically diagnosed diseases and 19,359 had one or more medically diagnosed diseases. After examining the final sample for the relevant quality requirements and possible anomalies, it was concluded that the distribution of municipality size, household size, internet use, gross annual income and education level closely resembled the Dutch population. However, it was also concluded that the final sample represented a population that was relatively old, slightly more female and somewhat more medically diagnosed as compared to the Dutch population. The socio-demographic characteristics of the final sample are displayed in Table 3.

**Table 3:** Sample description.

Age	Average of 53.7 years		
Gender	43.3% male	56.7% female	
Medical diagnosis	66.9% no	33.1% yes	
Household size	22.0% 1 person	47.0% 2 person	31.0% ≥ 3 person
Internet use	32.4% 0–4 h/day	31.5% 5–13 h/day	36.1% ≥ 14 h/day
Municipality population	45.4% 0–50,000	20.4% 50,001–100,000	34.2% ≥ 100,001
Gross annual income	37.9% 0–33,500 €	45.3% 33,501–67,000 €	16.8% ≥ 67,001 €
Education level	11.0% low	46.4% average	42.6% high

Although some socio-demographic characteristics of the final sample differed slightly from the Dutch population, it can be concluded that the final sample in general shows a reasonable resemblance to the Dutch population.

### Patient reported burden of disease ranking excluding comorbidities

As the expert reported burden of disease rankings are normally developed without taking comorbidities into account, this study first examines the distribution of different types of patients without comorbidities among the four segments of the Bloem & Stalpers model. Based on the proportions of patients in segment IV (see Fig. 1) of the Bloem & Stalpers model a first patient reported burden of disease ranking excluding comorbidities was developed. The most important findings, as displayed in Table 4, will be discussed in the rest of this article.

**Table 4:** Patient reported burden of disease ranking excluding comorbidities.

		Segment I	Segment II	Segment III	Segment IV	Total
Psychiatric diseases	N	71	22	53	168	314
	%	22.6%	7.0%	16.9%	53.5%	100.0%
Gastrointestinal diseases	N	108	28	47	188	371
	%	29.1%	7.5%	12.7%	50.7%	100.0%
Oncological diseases	N	119	59	12	177	367
	%	32.4%	16.1%	3.3%	48.2%	100.0%
Pain diseases	N	292	113	108	448	961
	%	30.4%	11.8%	11.2%	46.6%	100.0%
Rheumatological diseases	N	436	177	107	549	1,269
	%	34.4%	13.9%	6.4%	45.3%	100.0%
Neurological diseases	N	212	73	49	318	652
	%	32.5%	11.2%	13.4%	42.9%	100.0%
Pulmonary diseases	N	653	201	201	625	1,680
	%	38.9%	12.0%	12.0%	37.1%	100.0%
Urological diseases	N	30	14	8	30	82
	%	36.6%	17.1%	9.7%	36.6%	100.0%
Endocrinological diseases	N	383	92	84	306	865
	%	44.3%	10.6%	9.7%	35.4%	100.0%
Cardiovascular diseases	N	864	347	201	774	2,186
	%	39.5%	15.9%	9.2%	35.2%	100.0%
Dermatological diseases	N	122	43	31	93	289
	%	42.2%	14.9%	10.7%	32.2%	100.0%
Immunological diseases	N	11	2	0	6	19
	%	57.9%	10.5%	0.0%	31.6%	100.0%
Ophthalmological diseases	N	59	25	7	33	124
	%	47.6%	20.2%	5.6%	26.6%	100.0%
Total	N	3,360	1,196	908	3,715	9,179
	%	36.6%	13.0%	9.9%	40.5%	100.0%

This study shows that within the group of patients without comorbidities, psychiatric diseases (53.5%) have the largest percentage of patients in segment IV (see Fig. 1) of the Bloem & Stalpers model, followed by gastrointestinal diseases (50.7%), oncological diseases (48.2%), pain diseases (46.6%), rheumatological diseases (45.3%), neurological diseases (42.9%), pulmonary diseases (37.1%), urological diseases (36.6%), endocrinological diseases (35.4%), cardiovascular diseases (35.2%), dermatological diseases (32.2%), immunological diseases (31.6%) and ophthalmological diseases (26.6%). In the first six of these diseases, segment IV (see Fig. 1) of the Bloem & Stalpers model presented the largest share of patients. This means that the majority of patients with these diseases have difficulty accepting and controlling their personal health condition, resulting in relatively high burden of disease. For the other diseases, segment I (see Fig. 1) of the Bloem & Stalpers model presented the largest share of patients. This means that the majority of patients with these diseases is able to accept and control their personal health condition, resulting in relatively limited burden of disease. It should be noted that even patients with low ranked diseases could experience considerable difficulties accepting and controlling their disease, resulting in high burden of disease. In addition to the primary outcomes, some important observations, not presented in Table 3, need to be discussed. Firstly, this study shows that within the group of patients without comorbidities female patients are on average overrepresented in segment IV (see Fig. 1) of the Bloem & Stalpers model (56.2%). This indicates that female patients suffer more burden of disease relative to male patients. Secondly, this study shows that within the group of patients without comorbidities younger patients are on average overrepresented in segment IV (see Fig. 1) of the Bloem & Stalpers model (73.1%). This indicates that younger patients suffer more burden of disease relative to older patients.

### Patient reported burden of disease ranking including comorbidities

Although expert reported burden of disease rankings are generally developed without considering possible comorbidities, comorbidities are common in many diseases. Therefore, this study also explores the distribution of different types of patients with and without comorbidities among the four segments of the Bloem & Stalpers model. Based on the percentages of patients in segment IV (see Fig. 1) of the Bloem & Stalpers model, a second patient reported burden of disease ranking including comorbidities was established. The most noteworthy findings, as presented in Table 5, will be elaborated in the remainder of this article.

**Table 5:** Patient reported burden of disease ranking including comorbidities.

		Segment I	Segment II	Segment III	Segment IV	Total
Psychiatric diseases	N	177	87	148	898	1,310
	%	13.6%	6.6%	11.3%	68.5%	100.0%
Gastrointestinal diseases	N	459	203	264	1,676	2,602
	%	17.7%	7.8%	10.1%	64.4%	100.0%
Pain diseases	N	1,087	560	501	3,891	6,039
	%	18.0%	9.3%	8.3%	64.4%	100.0%
Neurological diseases	N	453	195	158	1,281	2,087
	%	21.7%	9.3%	7.6%	61.4%	100.0%
Rheumatological diseases	N	1,531	816	477	3,846	6,670
	%	22.9%	12.2%	7.2%	57.7%	100.0%
Oncological diseases	N	308	202	57	741	1,308
	%	23.5%	15.4%	4.4%	56.7%	100.0%
Urological diseases	N	198	116	51	410	775
	%	25.5%	15.0%	6.6%	52.9%	100.0%
Pulmonary diseases	N	1,512	654	535	2,987	5,688
	%	26.6%	11.5%	9.4%	52.5%	100.0%
Ophthalmological diseases	N	181	105	36	350	672
	%	26.9%	15.6%	5.4%	52.1%	100.0%
Dermatological diseases	N	257	125	76	482	940
	%	27.3%	13.3%	8.1%	51.3%	100.0%
Endocrinological diseases	N	932	329	258	1,556	3,075
	%	30.3%	10.7%	8.4%	50.6%	100.0%
Cardiovascular diseases	N	2,833	1,342	824	4,868	9,867
	%	28.7%	13.6%	8.4%	49.3%	100.0%
Immunological diseases	N	20	5	4	27	56
	%	35.8%	8.9%	7.1%	48.2%	100.0%
Total	N	9,948	4,739	3,389	23,113	41,189
	%	24.2%	11.5%	8.2%	56.1%	100.0%

This study suggests that within the group of patients with and without comorbidities, psychiatric diseases (68.5%) have the largest proportion of patients in segment IV (see Fig. 1) of the Bloem & Stalpers model, followed by gastrointestinal diseases (64.4%), pain diseases (64.4%), neurological diseases (61.4%), rheumatological diseases (57.7%), oncological diseases (56.7%), urological diseases (52.9%), pulmonary diseases (52.5%), ophthalmological diseases (52.1%), dermatological diseases (51.3%), endocrinological diseases (50.6%), cardiovascular diseases (49.3%) and immunological diseases (48.2%). In all of these diseases, segment IV (see Fig. 1) of the Bloem & Stalpers model presented the largest share of patients. This means that most patients with these diseases have problems accepting and controlling their personal health condition, generating high burden of disease. It should be mentioned that patients with low ranked diseases could still experience substantial difficulties accepting and controlling their disease, generating high burden of disease. Besides the main outcomes, some remarkable findings, not presented in Table 4, have to be discussed. Firstly, this study suggests that within the group of patients with and without comorbidities female patients are disproportionally represented in segment IV (see Fig. 1) of the Bloem & Stalpers model (60.9%). This shows that female patients experience more burden of disease compared to male patients. Secondly, this study suggests that within the group of patients with and without comorbidities younger patients are disproportionally represented in segment IV (see Fig. 1) of the Bloem & Stalpers model (66.0%). This shows that younger patients experience more burden of disease compared to older patients.

### Differences between burden of disease rankings

A comparison between both patient reported burden of disease rankings and the usual expert reported burden of disease rankings yields several notable differences. The most important findings, as displayed in Table 6, will be elaborated in the remainder of this article.

**Table 6:** Differences between burden of disease rankings.

	Expert reported	Patient reported*	Patient reported**
Oncological diseases	1	3	6
Cardiovascular diseases	2	10	12
Rheumatological diseases	3	5	5
Psychiatric diseases	4	1	1
Neurological diseases	5	6	4
Pulmonary diseases	6	7	8
Endocrinological diseases	7	9	11
Gastrointestinal diseases	8	2	2
Immunological diseases	9	12	13
Urological diseases	10	8	7
Ophthalmological diseases	11	13	9
Dermatological diseases	12	11	10
Pain diseases	–	4	3

*Patient reported burden of disease ranking without comorbidities.

**Patient reported burden of disease ranking with comorbidities.

This study shows that the differences between both patient reported burden of disease rankings are limited as the Spearman rank-order correlation coefficient is 0.89. The differences between the patient reported burden of disease rankings excluding comorbidities and the expert reported burden of disease rankings are more substantial as the Spearman rank-order correlation coefficient is 0.59. The differences between the patient reported burden of disease rankings including comorbidities and the expert reported burden of disease rankings are even bigger as the Spearman rank-order correlation coefficient is 0.30. Although some diseases, such as neurological diseases, show limited differences between rankings, many diseases show substantial differences. These differences are most pronounced in cardiovascular, psychiatric, gastrointestinal and pain diseases. The burden of cardiovascular diseases ranks high (2) on the expert reported burden of disease ranking, while the burden of these diseases ranks low (10) on the patient reported burden of disease ranking excluding comorbidities. This ranking is even lower (12) when including comorbidities. The burden of psychiatric diseases ranks moderately high (4) on the expert reported burden of disease ranking, while the burden of these diseases ranks highest (1) on the patient reported burden of disease rankings, both excluding and including comorbidities. The burden of gastrointestinal diseases ranks moderately low (8) on the expert reported burden of disease ranking, while the burden of these diseases ranks high (2) on the patient reported burden of disease rankings, both excluding and including comorbidities. The burden of pain diseases is still largely overlooked in the expert reported burden of disease ranking, while the burden of these diseases ranks moderately high (4) on the patient reported burden of disease ranking excluding comorbidities. This ranking is even higher (3) when including comorbidities.

## Discussion

In this study, patient reported burden of disease rankings are developed and compared with the usual expert reported burden of disease rankings. Prior research shows that patient reported and expert reported burden of disease could differ as patients tend to focus on subjective health experiences (quality of life) and experts tend to focus on mortality and disability (quantity of life)^8–10^. In accordance, this study shows that these differences are also reflected in the often used burden of disease rankings. In addition, this study shows that these differences are amplified when taking comorbidities into account. This study also shows that these differences are most pronounced in cardiovascular, psychiatric, gastrointestinal and pain diseases.

Earlier studies show that cardiovascular diseases are the worldwide number 1 cause of mortality despite declining mortality rates over last decades^25,26^. In accordance, this study shows that burden of cardiovascular diseases ranks high (2) on the expert reported burden of disease ranking. However, earlier studies also show that cardiovascular diseases are highly preventable and treatable due to the advent of surveillance, medication and treatment, resulting in relatively low patient reported burden of disease^27–29^. In accordance, this study shows that burden of cardiovascular diseases ranks low (10) on the patient reported burden of disease ranking excluding comorbidities. Earlier studies also show that common comorbidities of cardiovascular diseases (e.g., hypertension, high cholesterol, diabetes) may increase burden of disease^27–29^. In contrast, this study suggests that these comorbidities do not increase burden of disease as cardiovascular diseases rank lower (12) on the patient reported burden of disease ranking including comorbidities. A plausible explanation for this finding is that these comorbidities are also highly preventable and treatable^27–29^.

Previous research demonstrates that psychiatric diseases have a moderate mortality rate as an estimated 14.3% of annual deaths worldwide are attributable to these diseases^30,31^. In consonance, this study demonstrates that burden of psychiatric diseases ranks moderately high (4) on the expert reported burden of disease ranking. However, previous research also demonstrates that psychiatric diseases are difficult to remedy due to limited effectiveness of psychiatric treatment, generating particularly high patient reported burden of disease ^32,33^. In consonance, this study demonstrates that burden of psychiatric diseases ranks highest (1) on the patient reported burden of disease ranking excluding comorbidities. Previous research also demonstrates that comorbidities of psychiatric diseases (e.g., eating disorders, substance abuse) could increase burden of disease^32,33^. In consonance, this study demonstrates that more patients with psychiatric diseases accumulate in segment IV of the Bloem & Stalpers model when comorbidities are taken into account, indicating higher burden of disease. This also results in the highest rank (1) on the patient reported burden of disease ranking including comorbidities.

Preceding research suggests that gastrointestinal diseases have a moderately low mortality rate as deaths from these diseases declined to 10% of deaths worldwide over last decades^34–36^. In congruence, this study suggests that burden of gastrointestinal diseases ranks moderately low (8) on the expert reported burden of disease ranking. However, preceding research also suggests that gastrointestinal diseases are often not curable due to their chronic nature, generating high patient reported burden of disease^37,38^. In congruence, this study suggests that burden of gastrointestinal diseases ranks high (2) on the patient reported burden of disease ranking excluding comorbidities. Preceding research also suggests that comorbidities of gastrointestinal diseases (e.g., nausea, vomiting, constipation, diarrhoea, dysphoria, food avoidance) might increase burden of disease^37,38^. In congruence, this study suggests that more patients with gastrointestinal diseases accumulate in segment IV of the Bloem & Stalpers model when comorbidities are taken into account, indicating higher burden of disease. This also generates a high rank (2) on the patient reported burden of disease ranking including comorbidities.

Prior studies often consider pain diseases to be symptoms of other diseases and suggest that the modest relationship identified between pain diseases and increased mortality is nonsignificant^39–42^. Accordingly, this study indicates that burden of pain diseases is not included in the expert reported burden of disease ranking. However, prior studies also indicate that pain diseases are often complicated to treat due to their chronic nature and lack of clear underlying cause, generating high patient reported burden of disease^43,44^. Accordingly, this study indicates that burden of pain diseases ranks relatively high (4) in the patient reported burden of disease ranking excluding comorbidities. Prior studies also indicate that comorbidities of pain diseases (e.g., stress, depression, anxiety) could increase burden of disease^43,44^. Accordingly, this study suggests that more patients with pain diseases accumulate in segment IV of the Bloem & Stalpers model when comorbidities are taken into account, indicating higher burden of disease. This generates an even higher rank (3) on the patient reported burden of disease ranking including comorbidities.

Finally, this study found some remarkable results with regard to the representation of age and gender in segment IV of the Bloem & Stalpers model and the associated patient reported burden of disease rankings. Previous studies found that female patients could experience gender-bias resulting in less effective medical treatment and are prone to anxiety, stress and depression due to fluctuating ovarian hormone and estrogen levels, generating more burden of disease in female patients^45–47^. In consonance, this study found that female patients are overrepresented in segment IV of the Bloem & Stalpers model. This indicates that female patients experience more burden of disease than male patients. Previous studies also found that younger patients experience more difficulty accepting and controlling their diseases due to lack of life experience and limited acquiescence, generating more burden of disease in younger patients^48,49^. In consonance, this study found that young patients are overrepresented in segment IV of the Bloem & Stalpers model. This indicates that younger patients experience more burden of disease than older patients.

### Strengths and limitations

This study has several important strengths. The first strength of this study is the large size of the final sample, which increases the validity and reliability of the results. The second strength of this study is the large array of diseases represented in the final sample, which optimizes the breadth and scope of the results. The third strength of this study is the reasonable resemblance of the final sample to the socio-demographic characteristics of the Dutch population, which improves the generalizability and representativeness of the results. The fourth strength of this study is the use of the Bloem & Stalpers model and questionnaire, which provides a clear theoretical and conceptual foundation to the results. However, this study also has some limitations. The first limitation of this study is that the different diseases within the separate rankings cannot be easily compared among themselves, which might reduce the depth and detail of the results. The second limitation of this study is that there are still some diseases not presented in the final sample (e.g., cellulitis, eczema, renal insufficiency), which might limit the comprehensiveness and applicability of the results. The third limitation of this study is that the final sample is not necessarily representative of other populations than the Dutch population, which might compromise the generalizability of the results.

### Future research

This study has several implications for future research. The differences between expert reported and patient reported burden of disease found in this study imply that is important for future research to explore how clinicians could elicit and incorporate patient reported burden of disease in addition to expert reported burden of disease in clinical practice for the purpose of providing more effective treatment and improving treatment adherence. These differences also indicate that it is important for future research to explore how policymakers could evoke and utilize patient reported burden of disease in addition to expert reported burden of disease for the purpose of developing inclusive (public) health policies and tailored health services. These differences further suggest that it is important for future research to explore how economists could measure, predict and integrate patient reported burden of disease in addition to expert reported burden of disease for the purpose of more accurate resource allocation and comprehensive health economic evaluation. By pursuing these avenues for future research the existing knowledge on the elicitation, utilization and integration of patient reported burden of disease in addition to the usual expert reported burden of disease can be clarified and expanded.

## Conclusion

**Figure 2:**
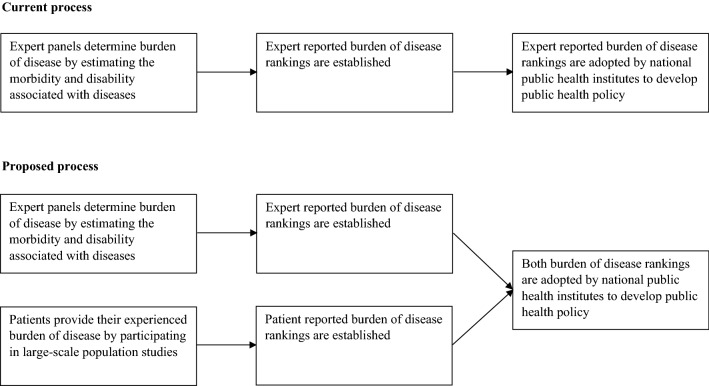
The current and proposed integration of burden of disease rankings in the development of public health policy.

Given the results of this study, it becomes evident that expert reported and patient reported burden of disease rankings could differ. The occurrence of comorbidities could amplify these potential differences between expert reported and patient reported burden of disease rankings even further. These differences are particularly pronounced in cardiovascular, psychiatric, gastrointestinal and pain diseases. Due to these differences, this study suggests that it can be beneficial to not only develop expert reported burden of disease rankings, but also to take in consideration the development of patient reported burden of disease rankings. This study introduces a valid and reliable way to develop such patient reported burden of diseases rankings, using the Bloem & Stalpers model. By developing both burden of disease rankings, decision-makers could gain insight in the overarching epidemiological implications of different diseases as well as the real-world needs, perspectives and experiences of patients with these diseases (see Fig. 2). This could facilitate a more comprehensive understanding of burden of disease and may improve decision-making on resource allocation, priority setting, cost-effectiveness evaluation, and service development in healthcare.

## Data Availability

The database used in this study is available from GfK, but restrictions apply regarding the availability of this database as it was used under license and is not publicly available. Nevertheless, the database can be made available by the corresponding author upon reasonable request and with permission of GfK.
